# UPLC-Q-TOF/MS-Based Serum and Urine Metabonomics Study on the Ameliorative Effects of Palmatine on *Helicobacter pylori*–Induced Chronic Atrophic Gastritis

**DOI:** 10.3389/fphar.2020.586954

**Published:** 2020-09-15

**Authors:** Xing Chen, Jianzhong Zhang, Ruilin Wang, Honghong Liu, Chunmei Bao, Shihua Wu, Jianxia Wen, Tao Yang, Ying Wei, Sichen Ren, Yuling Tong, Yanling Zhao

**Affiliations:** ^1^ Department of Pharmacy, Fifth Medical Center of PLA General Hospital, Beijing, China; ^2^ College of Pharmacy, Chengdu University of Traditional Chinese Medicine, Chengdu, China; ^3^ Center of Disease Control and Prevention, National Institute for Communicable Disease Control and Prevention, Beijing, China; ^4^ Integrative Medical Center, Fifth Medical Center of PLA General Hospital, Beijing, China; ^5^ Division of Clinical Microbiology, Fifth Medical Center of PLA General Hospital, Beijing, China

**Keywords:** palmatine, *Helicobacter pylori*, chronic atrophic gastritis, metabolomics, molecular mechanisms

## Abstract

**Objective:**

The main objective of this study was to investigate the ameliorative effects of Palmatine (Pal) on *Helicobacter pylori* (*H. pylori*) induced chronic atrophic gastritis (CAG)

**Method:**

Body function, serum biochemical indicators and histopathology were used to evaluate the pharmacodynamics of Pal on CAG rats. The target genes expression levels were verified and assessed by RT-PCR and immunohistochemistry (IHC). Moreover, UPLC-Q-TOF/MS analysis based on urine and serum was performed to identify the potential metabolites in the pathological process of CAG induced by *H. pylori*. Metabolic pathway analysis was performed to elucidate the metabolic network associated with Pal treatment of CAG.

**Results:**

Pal (10, 20, 40 mg/kg/day) significantly restored the body function of CAG rats, reduced the serum biochemical indicators, and maintained the integrity of the gastric mucosal epithelial barrier while alleviated gastric histological damage. Metabolomics analysis shows that the therapeutic effect of Pal on CAG involves 10 metabolites and 10 metabolic pathways, of which the Taurine and hypotaurine metabolism, Glycerophospholipid metabolism and Pentose and glucuronate interconversions are closely related to the gastrointestinal protection of Pal, and these metabolic pathways crosstalk with each other due to the internet hub of citric acid cycle.

**Conclusions:**

Metabolomics was used for the first time to identify potential biomarkers of CAG and to illuminate the therapeutic mechanism of Pal on CAG induced by *H. pylori*. The results provided a new insight for further research on CAG treatment.

## Introduction

Chronic atrophic gastritis (CAG) is a common disease of the digestive system which is considered a precursor condition for gastric cancer (GC). The rate of CAG progression to GC was as high as 2% per year during a 16 years of follow-up report (Lahner et al., 2015). It is understood that GC ranks fourth in the global incidence of various cancers worldwide and is the second cause of cancer-related deaths (Topi et al., 2020). *Helicobacter pylori* (*H. pylori*) is an obligate pathogen in the stomach, and the persistent inflammatory response caused by colonization is the strongest single risk factor for GC (Schulz et al., 2019). It has been recognized by the World Health Organization as a “definite carcinogen” since 1994, and the evidence for its role in gastric cancer (GC) development was expanded and updated in 2012 (Crafa et al., 2018). The risk of carcinogenic risk is closely related to the strain-specific bacterial components, host immune responses and effective drug intervention. Therefore, while focusing on the prevention of gastric cancer risk in people with *H. pylori* infection, delineating the drug treatment and mechanisms for gastric inflammation has profound significance for greatly reducing the incidence of CAG and GC.

Palmatine (Pal, **Figure 2A**) is an isoquinoline alkaloid from *Coptidis rhizoma* (Huanglian in Chinese), and is classified as an anti-inflammatory drug in Chinese Pharmacopeia (Tarabasz and Kukula-Koch, 2020). It can be used to relieve bacterial dysentery, gynecological inflammation and urinary tract-related surgical infections on clinic (Yokozawa et al., 2005; Wang et al., 2017). It has been the subject of scientific interest in recent years due to its multiple antibacterial, antiviral, and gastrointestinal protection biological activities (Li et al., 2018; Chen et al., 2020). However, the largely potential anti-gastritis mechanism of Pal remain unknown, and whether Pal can be safely and effectively used in clinical still needs more investigation.

Recently, omics techniques, such as transcriptomics, proteomics, and metabolomics have been widely used to identify potential disease markers and elucidate biologically related mechanisms. Metabolite profiling provides a clearer explanation of the relationship between drug efficacy, pharmacological effects, and metabolic pathways by analyzing specific biomarkers during disease and drug treatment. It has shown great potential in many life science fields, such as toxicology, disease diagnosis, drug mechanism research and natural product discovery (Wang et al., 2011). Previous studies have shown that *H. pylori* infection causes various metabolic alterations at lesional sites associated with disease transformation attribution. (Nishiumi et al., 2017). To the best of our knowledge, although the metabolites of Pal *in vivo* have been analyzed for quality evaluation or pharmacological activity (Wang et al., 2017), the analysis of Pal and its metabolitesmetabolite changes in *H. pylori*–induced gastritis individuals is currently lacking.

**Figure 1:**
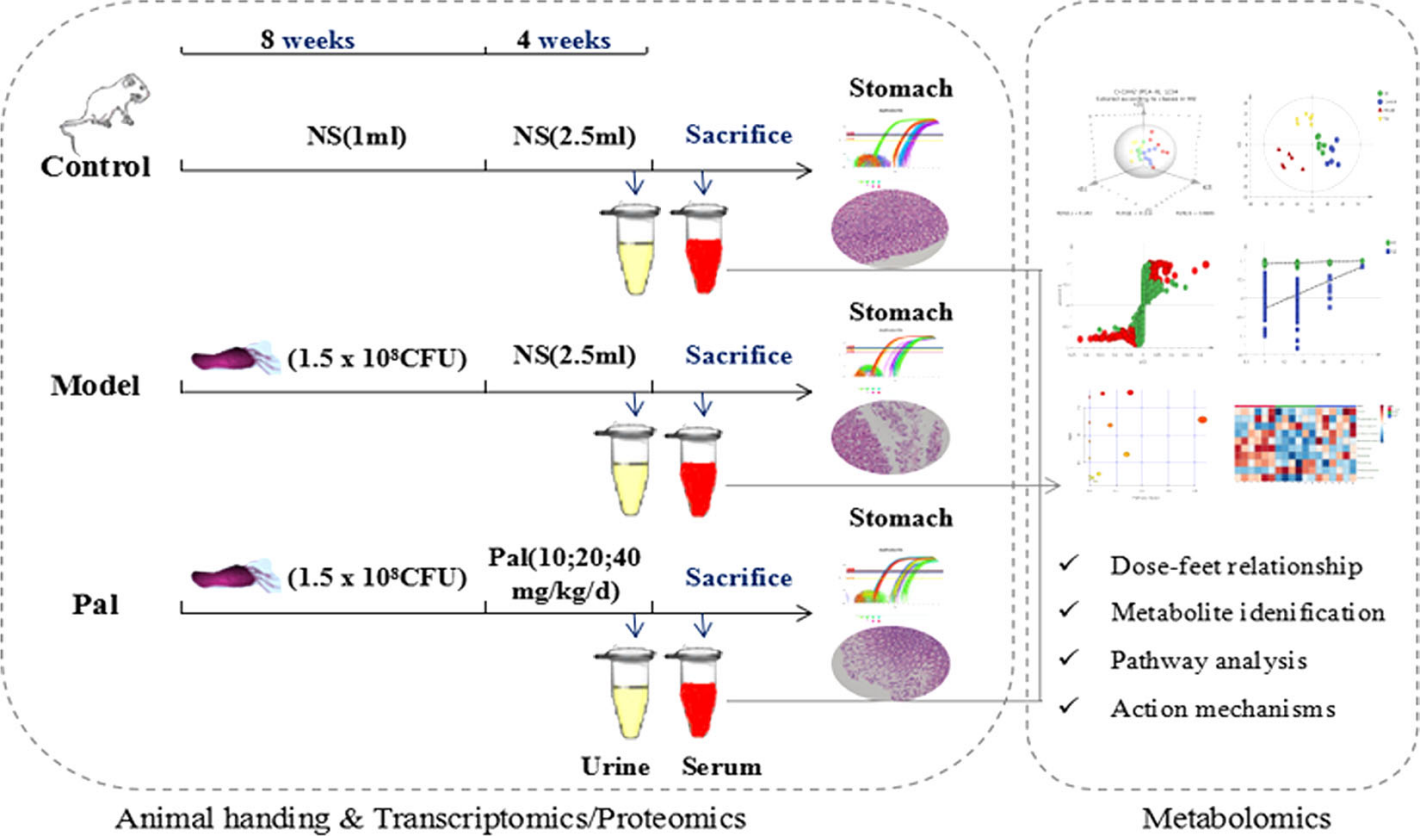
Scheme of the study.

All the considerations above prompted the application of metabolomics to comprehensively explore the gastrointestinal toxicity of Pal and its protective effect on *H. pylori*–induced CAG. In this study, serum biochemical indicators and pathological observations were used to evaluate theameliorative effect of Pal on CAG. UPLC-Q-TOF/MS metabolomics research was applied to obtain and analyze the changes of different metabolites in urine and serum during Pal therapy (**Figure 1**). Finally, it systematically clarified the modulatory properties of Pal on *H. pylori*–induced CAG, and further confirmed the feasibility of Pal for treatment of *H. pylori*–induced CAG.

## Materials and Methods

### Chemicals and Reagents

Pal standard (C_21_H_25_NO_4_, purity > 90%, Cat. No.10605-02-4) was purchased from Chroma Biotechnology Co. Ltd (Chengdu, China). Dissolved it into normal saline (NS 0.9%) and diluted to the corresponding concentration for later use.

### H. pylori Strains and Growth Condition

*H. pylori* strain was kindly provided by Prof. Jianzhong which was isolated from CAG patient (No. ZCDC111001). Columbia agar plats (Thermo Fisher Scientific Co., Ltd., Beijing, China) was used for bacterial propagation and shake for 48–72 h under the microaerobic conditions (6% O2, 7.2% CO2, 7.1% H2, 79.7% N2) of Low Oxygen Cell Culture Jar (UNITECH Co., Ltd., Guangzhou, China).

### Animals

Male Sprague Dawley (SD) rats (180 ± 20 g) were purchased from Sibeifu Biotechnology Co., Ltd. (Permission No. SCXK (jing) 2016-0002, Beijing, China). Rats were adapted the specific environment (humidity: 55% ± 5%, temperature: 25°C ± 0.5°C and 12-h/12-h light/dark cycle) and given sufficient sterile food and water for 2 weeks. All experimental procedures have been reviewed and approved by the Animal Ethical and Experimental Committee of the Fifth Medical Center of PLA General Hospital (Approval ID: IACUC-2018-010) and strictly implemented in accordance with the “Guide for the Care and Use of Laboratory Animals” (https://grants.nih.gov/grants/olaw/guide-for-the-care-and-use-of-laboratory-animals.pdf).

### Induction of CAG

CAG model was induced by *H. pylori* as previously described (Werawatganon, 2014). Live bacteria were collected and adjusted to 1.5 × 10^8^ colony forming units (CFU)/ml with normal saline (NS). Rats were administered with *H. pylori* strains at 1 day intervals to induce CAG model. Hematoxylin and eosin (H & E) staining and a rapid urease kit were used to evaluate the inflammation of stomach tissue at 8 weeks of infection. After CAG model was successfully duplicated, animals were randomly assigned into five groups (n = 6/group): Control group, Model group, Pal group (PalL PalM, PalH = 10, 20, 40 mg/kg/day) respectively. A normal diet was maintained during the 4 weeks of Pal treatment. In this study, the doses of Pal was referenced to previous experiments (Jung et al., 2014).

### Sample Collection and Preparation

At the end of the experiment, each rat was put into a metabolic cage (1 per cage) to collect 24-h urine and then sacrificed rats to obtain blood from aorta abdominalis.

Collected the gastric tissue and cut along the large curvature of the stomach, soaked in 10% neutral formalin buffer and embedded in paraffin to observe the pathological changes. The blood was centrifuged at 3.5 × 10^3^ rpm for 12 min to separate the serum and transferred to −8°C for storage before biochemical parameters and metabolomics analysis.

### Histopathological Analysis

Cuted 3–5μm thick paraffin slice of stomach tissue. H & E staining was performed for highlighting the pathological damage and inflammation of stomach tissues. The test result was observed with a light microscope (200 ×, 400 × magnifications).

### Measurement of Cytokine Concentrations

Serum was stored at −80°C before to cytokine determination. The levels of Pepsinogen I (PG I), Pepsinogen II (PG II), Gastrin17 (G-17) and Ghrelin were quantified by ELISA kits (MLBIO biotechnology Co., Ltd., Shanghai, China) according to the protocols provided by the manufacturer. Synergy H1 Hybrid Reader (Biotek, Winooski, VT, USA) was used to measure the absorbance at 450 nm.

### Quantitative RT-PCR

Total RNA was extracted from stomach tissues by using Trizol reagent (Nordic Bioscience, Beijing, China) and then converted into cDNA by reverse transcription kit (Promega, Madison, USA). RT-PCR was selected SYBR Green PCR Master Mix (Nordic Bioscience, Beijing, China) and executed on QuantSTUDIO TM 6 Flex System (Applied Biosystems, Foster City, CA, USA). The level of mRNA was normalized to GAPDH expression based on 2^-ΔΔCT^ method. The primers sequences of IFN-γ, Ghrelin, Epithelial cadherin (E-Cadherin), Desmoglein 2 (DSG 2), Zonula occludens-1 (ZO-1) and Mucin 1(Muc1) were listed in **Table 1**.

**Table 1:** Primers sequences.

Gene	Forward primer	Reverse primer
Rat IFN-γ	ACAACCCACAGATCCAGCACAAAG	GCTTCCTTAGGCTAGATTCTGGTGAC
Rat Ghrelin	CCAGAAAGCCCAGCAGAGAAAGG	ACATCGAAGGGAGCATTGAACCTG
Rat E-Cadherin	CCTACAATGCTGCCATCGCCTAC	GGGTAACTCTCTCGGTCCAGTCC
Rat Dsg2	CGGAGTGTCTGTGCGGATGC	GGCTGGTCAATGATGGAGAAGGTG
Rat ZO-1	CGCAGCCAGTTCAAACAAAGTTCC	GCAACATCAGCAATCGGTCCAAAG
Rat Muc1	GCCCATCTCTCCCACCATCCC	GGAAGACCGCTGTGCTGTAGTAAG

### Immunohistochemistry/Immunofluorescence

Paraffin sections of stomach tissues were stained with anti-E-Cadherin, anti-DSG 2, anti-ZO-1, anti-Muc1 Ab (**Table 2**) for IHC/immunofluorescence (IF), and lastly examined using NIS Elements Imaging Software Version 4.0 (Olympus, Japan).

**Table 2:** Antibodies and other reagents.

Antibodies	Dilution rate	Manufacturers	Cat.No.
**For IHC**			
Rabbit anti-Rat Desmoglein2/DSG2	1/250–1/500	Abcam	ab150372
Rabbit anti-Rat ZO-1 tight junction	1/500	Abcam	ab221547
Rabbit anti-Rat E-Cadherin	1:50–1:200	Cell signaling technology	14472
**For IF**			
Rabbit anti-Rat Muc1	1/500	Abcam	ab45167

### Metabolic Profiling

The serum and urine samples were thawed at room temperature prior to analysis. Added three times methanol to serum or urine and centrifuged at 12,000 rpm for 12 min to precipitate the protein. Collected the supernatant filtrate with nylon filter. Ten microliter of each sample was mixed to prepare quality control (QC), and the remaining samples were used for UPLC-Q-TOF/MS testing.

An Agilent 1290 series UHPLC system (Agilent Technologies, Santa Clara, CA, USA) system was used for serum and urine metabolic profiling analysis. ZORBOX RRHD C18 analytical column (2.1 mm × 100 mm, 1.8 μm., Agilent Technologies, Santa Clara, CA, USA) was used for sample separation at 30°C. The injection volume was 4 μl and flow rate was 0.30ml/min. Solvent A (0.1% formic acid in acetonitrile) and solvent B (0.1% formic acid in water) were mixed into a gradient mobile phase under a 25-min gradient program: 0–1.0 min (95% A); 1.0–9.0 min (95%–60% A); 9.0–19.0 min (60%–10%); 19.0–21.0 min (10%–0% A); 21.0–25.0 min (100% B). Each sample is injected only once, a QC sample and a blank were injected after every 10 samples to ensure the stability and repeatability of the system. Both positive and negative mode electrospray ionization sources (ESI) were used (Electrospray capillary voltage: 4.0kV in ESI+ and 3.5 kV in ESI-; Mass range: 50 to 1,200 m/z; Gas flow:13 L/min; Nebulizer: 20 psi; Sheath gas temperature: 275°C; Sheath gas flow: 12 L/min; Nozzle voltage: 2,000V) in this study.

### Data Extraction and Multivariate Analysis

Sample data was extracted by using MassHunter Profinder software (version B.06.00; Agilent, Santa Clara, CA, USA) for analysis and comparison. Data was normalized with MetaboAnalyst 4.0 and then SIMCA-P V14.1 (Umetrics, Umea, Sweden) was used for principal component analysis (PCA) and orthogonal-partial least squares-discriminant analysis (OPLS-DA). The potential biomarkers were identified based on precise molecular weight in the Human Metabolome Database (HMDB) and METLIN (http://metlin.scripps.edu/) database. MetaboAnalyst 4.0 (http://www.metaboanalyst.ca/) was visualized enrichment pathway of potential markers.

### Statistical Analysis

Statistical analysis was performed by SPSS 20.0 software program (Chicago, IL, USA) using one-way ANOVA followed by t-test. GraphPad Prism software (version 6.02; Inc., San Diego, USA) was used to visualize the results. *P* < 0.05 was considered statistically significant.

## Results

### Pal Attenuated Pathological Damage and Inflammation in H. pylori–Induced CAG

**Figure 2:**
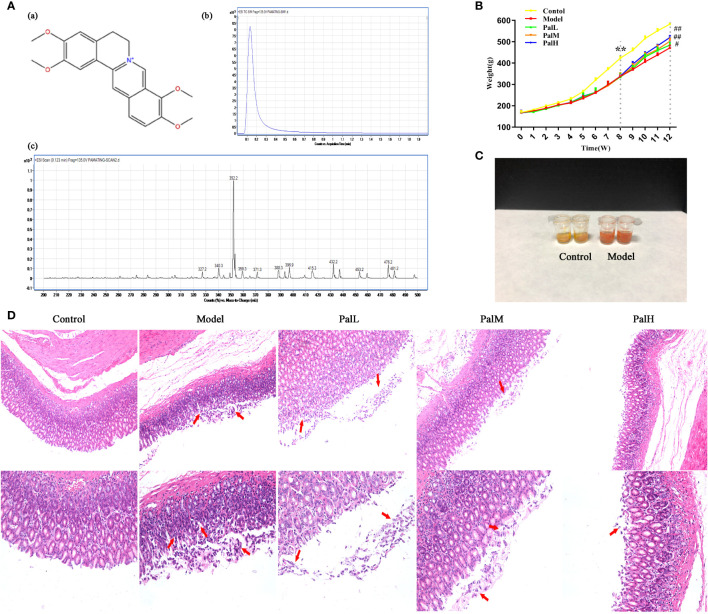
Pal attenuated pathological damage and inflammation in *H. pylori*–induced chronic atrophic gastritis (CAG). **(A)** Chemical structure (a), TIC (b) and Scan (c) chromatogram of Pal. **(B)** Weight of SD rats (n=6). **(C)** Rapid urease test of stomach tissues. **(D)** Morphology and hematoxylin and eosin (H & E) staining of rats stomach (200 × and 400 × magnification). **P < 0.01 vs control group. ^#^ *P* < 0.05 and ^##^ *P* < 0.01 vs model group. PalL (10 mg/kg); PalM: (20 mg/kg); PalH: (40 mg/kg).

During *H. pylori* infection, rats infected with *H. pylori* developed symptoms of inappetence, diarrhea, and the weight gain trend of the model group was markedly slower than control group during the whole experiment (**Figure 2B**). In addition, the positive results of the rapid urease kit proved the reliability of the model (**Figure 2C**). The pathological result showed that the epithelial cells of gastric mucosa in control group were arranged neatly without shedding, and the morphology of submucosal was normal; model group was characterized by thin mucosa and partial mucosa shedding, submucosal edema and eosinophil infiltration in the stomach, atrophy of glands at the junction of the anterior and posterior stomach and reduction of inherent glands (**Figure 2D**). Those changes were similar to CAG patients caused by *H. pylori* in clinic. After 4 weeks of Pal intervention, the body weight of CAG rats gradually recovered, and the pathological gastric mucosal damage was significantly reduced (**Figure 2D**), suggesting that Pal had a significant protective effect on CAG induced by *H. pylori*.

### Pal Improved Biomarkers in Serum of H. pylori–Induced CAG

**Figure 3:**
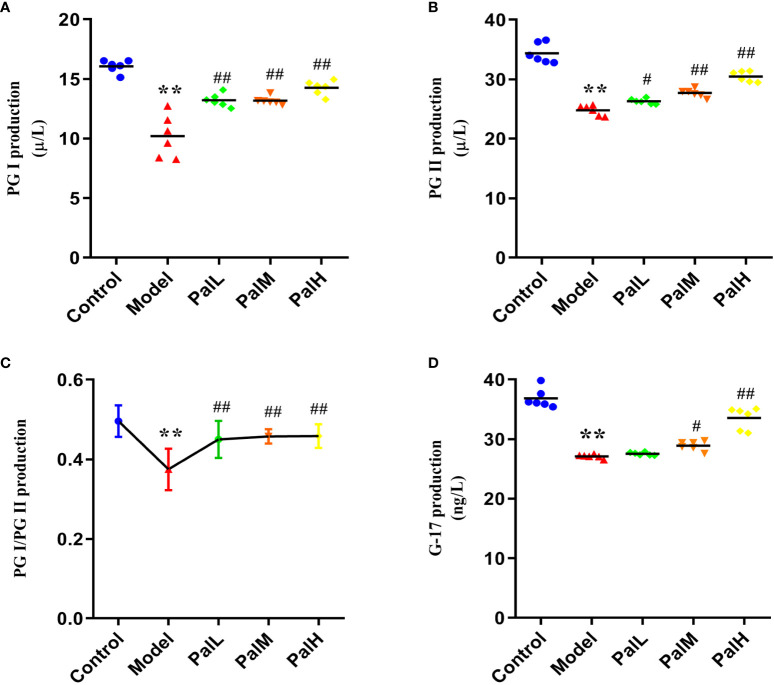
Pal improves biomarkers in serum of *H. pylori*–induced chronic atrophic gastritis (CAG). **(A–D)** PG I **(A)**, PG II **(B)**, PG I/PG II **(C)**, and G-17 **(D)** production in serum was measured by ELISA (n=6). **P < 0.01 vs control group. ^#^ *P* < 0.05 and *^##^P *< 0.01 vs model group. PalL (10 mg/kg); PalM: (20 mg/kg); PalH: (40 mg/kg).

To clarify the activities of several specific markers of *H. pylori*–induced CAG rats, the serum supernatant levels of serum PG I, PG II, and G-17 were estimated. As showed in **Figure 3**, PG I, PG II, PG I/PG II, and G-17 decreased markedly in *H. pylori*–induced model group compared to control group. Conversely, PG I, PG II, and G-17 significantly increased after Pal administration, and the above biomarkers in the PalH group maintained higher levels compared with the PalL group (**Figures 3A–D**). In addition, Pal reversed the decrease in the ratio of PG I/PG II induced by *H. pylori* in the model group, which was more obvious in PalH group (**Figure 3C**). Altogether, these results *in vivo* implied that the activities of several specific markers, including PG I, PG II, and G-17, and the ratio of PG I/PG II, could get dose-dependently improved by Pal intervention.

### Pal Relieved the Release Disorder of Ghrelin by Inhibiting IFN-γ

**Figure 4:**
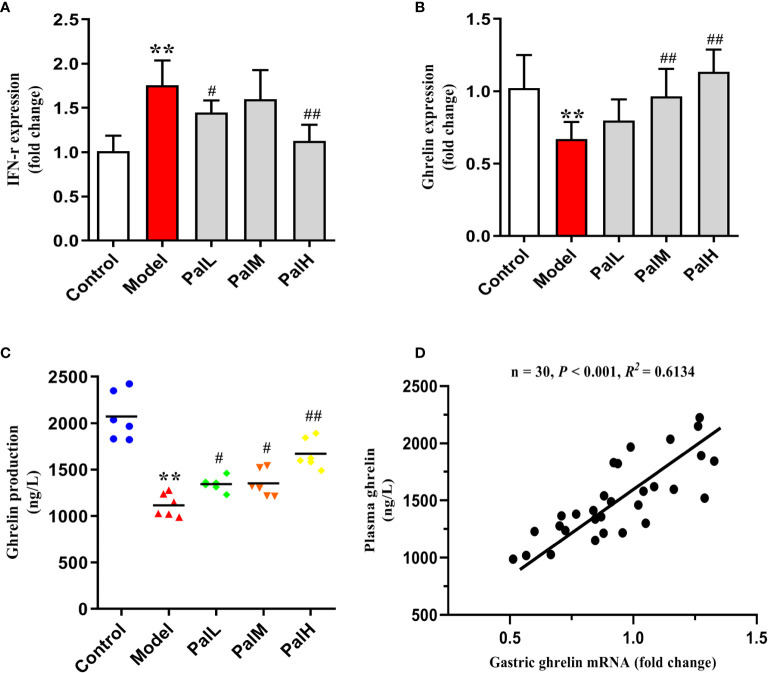
Pal relieved the release disorder of ghrelin by inhibiting IFN-γ. **(A, B)** IFN-γ and ghrelin mRNA in gastric mucosa were analyzed by RT-PCR (n=6). **(C)** ghrelin production in serum was measured by ELISA (n=6). **(D)** Pearson correlation coefficient analysis of ghrelin expression in gastric mucosa and serum. **P < 0.01 vs control group. ^#^ *P* < 0.05 and ^##^ *P* < 0.01 vs model group. PalL (10 mg/kg); PalM: (20 mg/kg); PalH: (40 mg/kg).

To test whether the CAG rat model induced by *H. pylori* was IFN-γ associated inflammation and would further change the expression of ghrelin, the expression of IFN-γ and ghrelin in stomach and serum were evaluated by RT-PCR and ELISA respectively. Results were presented in **Figure 4**. Evidently, the significant increase of IFN-γ expression in stomach of *H. pylori* infected rats was accompanied by a decrease in the expression of ghrelin, while the concentration of ghrelin in serum was also significantly reduced (**Figures 4A–C**). Accordingly, these findings indicated that IFN-γ inflammatory response and impaired production of ghrelin in the stomach are associated with CAG induced by *H. pylori*, which ultimately leads to a decrease in the concentration of serum ghrelin (**Figure 4D**). However, Pal could dose-dependently reduced the level of IFN-γ gene and alleviated the release of ghrelin. Consequently, the above results suggested that Pal could restore ghrelin expression by inhibiting IFN-γ and had anti-inflammatory properties in CAG induced by *H. pylori*.

### Pal Protected Mucosal Integrity by Modulating Epithelial Junctions and Mucins

**Figure 5:**
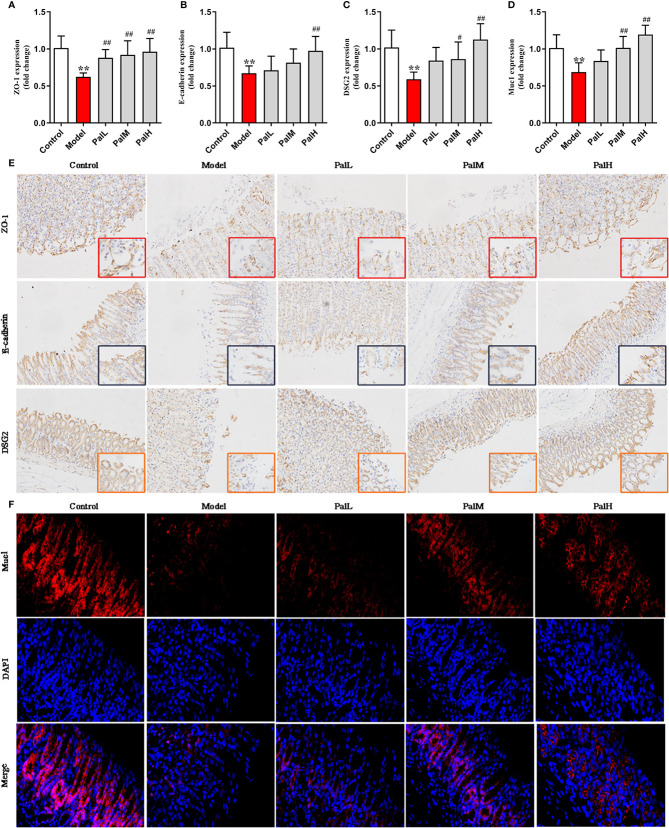
Pal protected mucosal integrity by modulating epithelial junctions and mucins. **(A–D)** ZO-1 **(A)**, E-cadherin **(B)**, Desmoglein 2 (DSG 2) **(C)** and Muc1 **(D)** mRNA in gastric mucosa were analyzed by RT-PCR (n=6). **(E)** Representative immunohistochemical staining images in gastric mucosa showing ZO-1, E-cadherin, DSG2 expressing (200 × and 400 × magnification). **(F)** Representative immunofluorescence staining images in gastric mucosa showing Muc1 expressing (400 × magnification). **P < 0.01 vs control group. ^#^ *P* < 0.05 and ^##^ *P* < 0.01 vs model group. PalL (10 mg/kg); PalM: (20 mg/kg); PalH: (40 mg/kg).

To further evaluated the protective effect of Pal on the gastric mucosa of CAG rats induced by *H. pylori*, the mRNA expression of ZO-1, E-cadherin, DSG2, Muc1 were detected using RT-PCR. The results show that the expression of the above mentioned mRNA in the model group was significantly downregulated compared with the control group. However, Pal significantly reversed the inhibitory effect of *H. pylori* (**Figures 5A–D**).

Subsequently, the protein expression of ZO-1, E-cadherin, DSG2, Muc1 were measured to evaluate the integrity on gastric mucosa epithelium. The results showed that all proteins were reduced in model group, indicating that the gastric mucosa epithelium structure was disrupted. In contrast, Pal promoted the expression of ZO-1, E-cadherin, DSG2 and increased the fluorescence intensity of Muc1, and there was a statistically significant difference between the PalH group and the model group (**Figures 5E, F**). Therefore, Pal could protect mucosal integrity by modulating epithelial junctions and mucins, and PalH group was selected for subsequent metabolomics analysis.

### Serum Metabolomics Analysis

**Figure 6:**
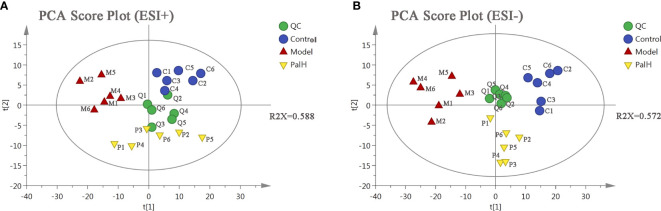
The principal component analysis (PCA) score plot of serum metabolomics analysis. **(A)** ESI+ model. **(B)** ESI- model. PalH: (40 mg/kg).

PCA is an unsupervised pattern recognition approach used to reduce the dimension of UPLC-MS data and to disclose intrinsic clustering of samples. In this study, PCA was used to summarize and distinguish the metabolic phenotypes and metabolites between control, model, and PalH group in both ESI+ and ESI- models. QC can confirm whether the systematic error of the experiment is within the controllable range. The cluster analysis of QC suggested that the method was stability and repeatability. The PCA score plots showed that clear separations were observed among the clusters of the control, model and PalH group in both ESI+ and ESI− modes (**Figure 6**), which indicated *H. pylori*–induced remarkable changes in serum endogenous metabolites and Pal could restore the metabolic profiling of serum in CAG rats. The R^2^X of ESI+ and ESI− PCA models confirmed the credibility of these two models. Thus, the CAG model was successful, and could be used to explore the effects of Pal on CAG induced by *H. pylori*. Subsequently, Summarized the influence of variables on the model according to the corresponding loading scores plots from PCA, multivariate analysis was used to explore specific differential metabolites.

**Figure 7:**
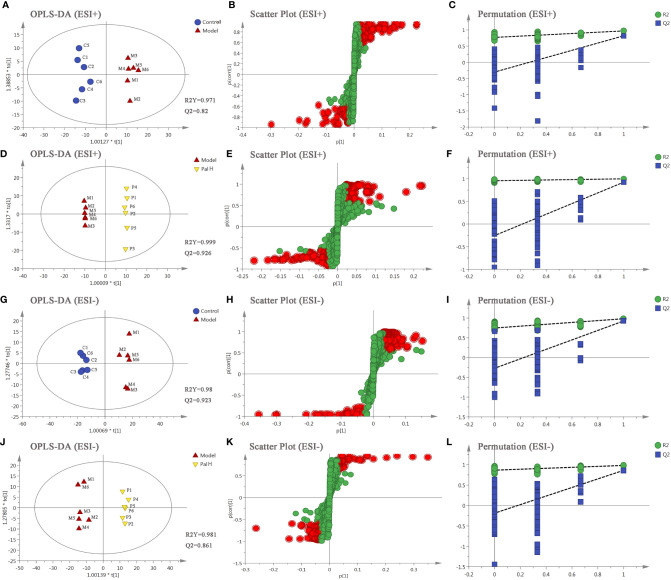
The orthogonal-partial least squares-discriminant analysis (OPLS-DA) score plots, S-plots and 100-permutation test of serum metabolomics analysis. **(A–F)** ESI+ model. **(G–L)** ESI- model. PalH: (40 mg/kg).

**Figure 8:**
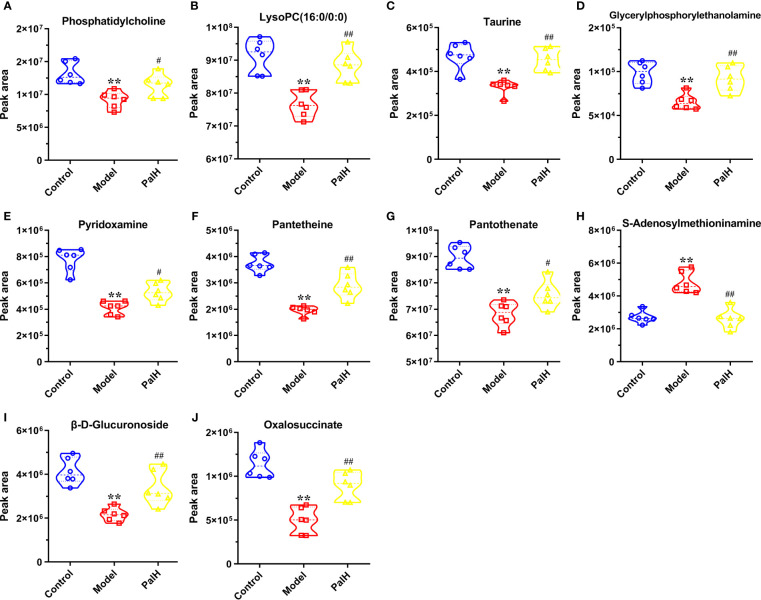
Potential metabolites changes in *H. pylori* - induced CAG with PalH treatment. **(A)** Phosphatidylcholine. **(B)** LysoPC(16:0/0:0). **(C)** Taurine. **(D)** Glycerylphosphorylethanolamine. **(E)** Pyridoxamine. **(F)** Pantetheine. **(G)** Pantothenate. **(H)** S-Adenosylmethioninamine. **(I)** β-D-Glucuronoside. **(J)** Oxalosuccinate. **P < 0.01 vs control group. ^#^ *P* < 0.05 and ^##^ *P* < 0.01 vs model group. PalH: (40mg / kg).

The differential metabolites from control vs model group and model vs PalH group were identified in OPLS-DA mode respectively. The relevant parameter of OPLS-DA model and permutation tests (n=100) demonstrated that the modes had good predictive capabilities. Obvious classifications were observed among control, model and PalH groups in both of ESI+ and ESI- modes. Metabolites that meet a threshold of VIP ≥ 1.0 and | *P* (corr) | ≥ 0.58 in the corresponding S-plot were considered potential candidates (**Figure 7**). Combined the potential candidates obtained in ESI+ and ESI−. Based on ANOVA analysis (*P* < 0.05), candidates with significant changes were identified as biomarkers in the METLIN and Metaboanalyst databases. Finally, three biomarkers were screened in serum, and relevant information and variation trends between groups were listed in **Table 3** and **Figure 8**.

**Table 3:** Identified metabolites of serum and urine.

No	Source	Compound name	Formula	Query mass	Monoisotopic_mass	R.T. (min)	Pathway
1	Serum(ESI+)	Phosphatidylcholine	C_46_H_82_NO_7_P	792.5951	791.5829	7.14	Glycerophospholipid metabolism
2	Serum(ESI-)	LysoPC(16:0/0:0)	C_24_H_50_NO_7_P	496.3396	495.3325	15.35	Glycerophospholipid metabolism
3		Taurine	C_2_H_7_NO_3_S	124.0073	125.0147	0.89	Taurine and hypotaurine metabolism; Primary bile acid biosynthesis
4	Urine(ESI+)	Glycerylphosphorylethanolamine	C_5_H_14_NO_6_P	216.0631	215.0559	5.51	Glycerophospholipid metabolism
5		Pyridoxamine	C_8_H_12_N_2_O_2_	169.0971	168.0898	2.35	Vitamin B6 metabolism
6		Pantetheine	C_11_H_22_N_2_O_4_S	279.1318	278.1300	5.39	Pantothenate and CoA biosynthesis
7		Pantothenate	C_9_H_17_NO_5_	220.1172	219.1107	2.91	Pantothenate and CoA biosynthesis
8		S-Adenosylmethioninamine	C_14_H_23_N_6_O_3_S	355.1565	355.1552	0.85	Cysteine and methionine metabolism; Arginine and proline metabolism
9	Urine(ESI-)	β-D-Glucuronoside	C_14_H_18_O_7_	297.0975	298.1053	2.65	Pentose and glucuronate interconversions
10		Oxalosuccinate	C_6_H_6_O_7_	189.0038	190.0114	2.72	Citrate cycle (TCA cycle)

### Urine Metabolomics Analysis

**Figure 9:**
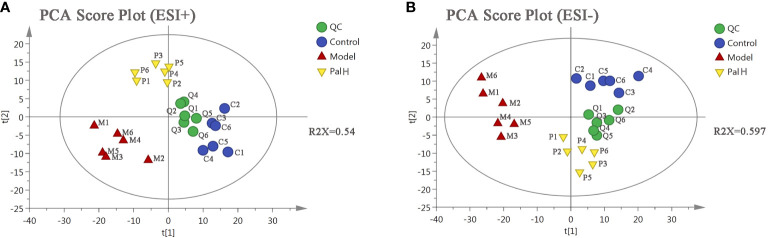
The principal component analysis (PCA) score plot of urine metabolomics analysis. **(A)** ESI+ model. **(B)** ESI- model. PalH: (40 mg/kg).

To further explored the overall metabolism in the urine of the CAG, PCA approach was applied to distinguish the metabolic phenotypes and metabolites between control, model, and PalH groups. The R^2^X of ESI+ and ESI− in PCA models confirmed the reliability of these two models. The PCA score plots indicated remarkable changes of endogenous metabolites in urine induced by *H. pylori* and Pal could restore the metabolic profiling of urine in CAG rats (**Figure 9**).

**Figure 10:**
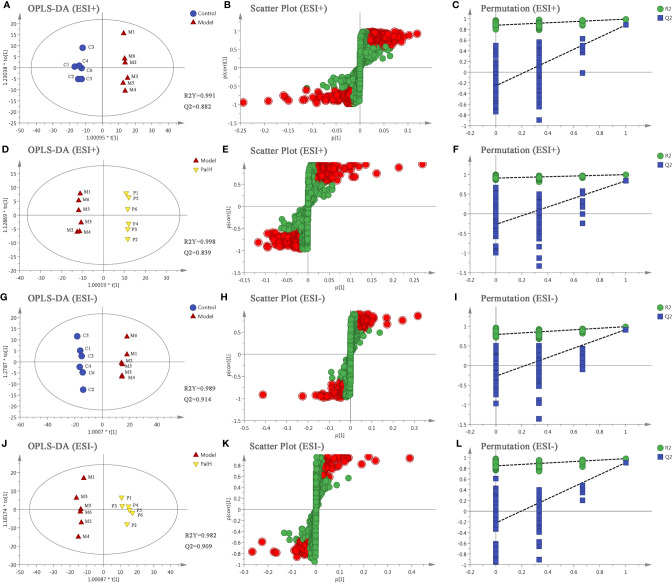
The orthogonal-partial least squares-discriminant analysis (OPLS-DA) score plots, S-plots and 100-permutation test of serum metabolomics analysis. **(A–F)** ESI+ model. **(G–L)** ESI- model. PalH: (40 mg/kg).

OPLS-DA score plots showed that the metabolic profile of the model group deviated from control group, and PalH group in both of ESI+ and ESI- modes, suggesting that significant biochemical changes were induced by CAG. The relevant parameter of OPLS-DA mode and permutation tests (n = 100) demonstrated that the modes had good explanatory and predictive capabilities (**Figure 10**). Combined the potential candidates obtained in ESI+ and ESI-, and further determined biomarkers by using ANOVA analysis. Finally, the seven biomarkers in urine were summarized. **Table 3** and **Figure 8** listed the corresponding formulations, quality (m/z), retention time and change trend of each group.

### Pathway Analysis of Pal Treatment

**Figure 11:**
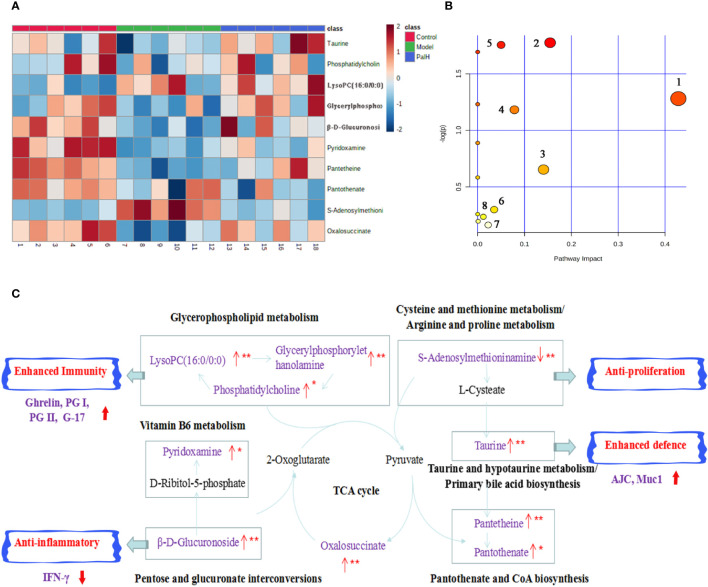
Pathway analysis of Pal treatment. **(A)** The heatmap of 10 potential metabolites. **(B)** Metabolomic pathway construction of the metabolic pathways involved in the effects of Pal on chronic atrophic gastritis (CAG). **(C)** Signaling networks associated with the differentially expressed metabolic pathways. Red represents the detected metabolite.

The hierarchical cluster analysis heatmap generated according to the relative concentration of metabolic markers in serum and urine can intuitively displayed the differences in metabolic profiles between different samples (**Figure 11A**). MetaboAnalyst 4.0 was performed for pathway analysis to visualize the affected metabolic pathways in CAG rats. As shown in **Table 4** and **Figure 11**, Taurine and hypotaurine metabolism, Glycerophospholipid metabolism, Pentose and glucuronate interconversions were considered as the crucial signaling pathways in CAG and Pal treatment. In addition, the related metabolic pathways crosstalk with each other by the citric acid (TCA) cycle. The match status, *P* value, log(P) and impact of each pathway were listed in **Table 4**.

**Table 4:** Results of integrating enrichment analysis of biomarkers with MetaboAnalyst 4.0.

No	Pathway	Match status	Raw *p*	− log(p)	Impact
1	Taurine and hypotaurine metabolism	1/8	0.27773	1.2811	0.42857
2	Glycerophospholipid metabolism	3/36	0.16941	1.7754	0.15471
3	Pentose and glucuronate interconversions	1/18	0.52028	0.65339	0.14062
4	Vitamin B6 metabolism	1/9	0.3066	1.1822	0.07843
5	Pantothenate and CoA biosynthesis	2/19	0.1728	1.7556	0.05
6	Cysteine and methionine metabolism	1/33	0.7417	0.29881	0.03514
7	Primary bile acid biosynthesis	1/46	0.84974	0.16283	0.02239
8	Arginine and proline metabolism	1/38	0.79016	0.23552	0.01212
9	Citrate cycle (TCA cycle)	1/20	0.18215	1.7029	0.00

## Discussion

Huang Lian has been applied to endocrine system and gastrointestinal diseases in clinic for thousands of years (Zou et al., 2017; Ma et al., 2019). More and more studies regarding the effects of the active ingredients in HuangLian on gastrointestinal diseases is available. Previous studies have shown that Pal and berberine inhibited the proliferation of gastric cancer cells (MGC803) in a dose-dependent manner and the inhibitory effect more significantly in combination with berberine (Zhao and Zhang, 2011). Jung J et al. demonstrated the antibacterial activity of Pal and its protective effect on gastric injury through the *H. pylori* colony formation experiment and the hydrochloric acid/ethanol induced gastric ulcer model, and the efficacy is better than berberine (Jung et al., 2014). Moreover, Pal was confirmed to have a clear antibacterial activity against *H. pylori* in a molecular docking simulation experiment (Zhou et al., 2017). Therefore, Pal is considered to be a promising non-antibiotic drug candidate for the treatment of *H. pylori*–induced CAG.

*H. pylori*–induced gastritis is one of the optimized models used to study CAG. As in humans, weight loss, inappetence, diarrhea, and pathological changes in rats are usually consistent symptoms of CAG (Kishikawa et al., 2020). Given that the intractable characteristic and high morbidity of CAG, UPLC-Q-TOF/MS method based on serum combined with urin metabolomics was firstly investigated in this study to clarify the protective effect of Pal on CAG and attempted to clarify the mechanisms of action.

Gastric infections due to *H. pylori* overgrowth are associated with reduced fundic ghrelin and G-17 expression and increased IFN-γ production (Strickertsson et al., 2011). Ghrelin plays an essential role in the mechanism of gastric mucosal defense, the reduction of ghrelin production in the fundic is the main reason for the decrease of serum ghrelin concentration, and it is closely related to the ratio of PG I/PG II, in which the PG I/PG II < 3 is considered a reliable sign of severe atrophic gastritis. PG I is secreted only by secretory glands of the corpus mucosa, PG II is synthesized by gastric antrum and duodenum, and G-17 is only expressed by G cells of the antral mucosa. Thus the combination of the results of the PG I, PG II, PG I/PG II and G-17 test can more comprehensively and accurately detect the presence of CAG (Osawa et al., 2005; Zagari et al., 2017). In this study, the model group had high levels of pro-inflammatory cytokines IFN-γ, low levels of ghrelin, G-17, PG I, PG II, and PGI/PGII ratios. However, the levels of biomarkers and proinflammatory cytokines was decreased in Pal treatment revealed that Pal could effectively ameliorate CAG triggered by *H. pylori*.

CAG induced by *H. pylori* is triggered by the destruction of gastric mucosal epithelial barrier function, which further leads to excessive response of immune cells to microbiota. The gastrointestinal mucosa can protect the digestive tract from harmful substances and is an important barrier to maintain environmental stability in the digestive tract (Zhang et al., 2014). This mucosal barrier is predominantly composed of apical junctional complex (AJC) and mucins. AJC include tight junctions (TJs), adherens junctions (AJs) and desmosomes. Muc1 is a prototypical member of the membrane bound mucin subfamily. In this study, the expression and distribution of TJ proteins (ZO-1), AJ proteins (E-cadherin), desmosome protein (DSG2), and Muc1 in gastrointestinal epithelial cells infected with *H. pylori* were systematically investigated. The mRNA and protein expression of AJC and Muc1 were significantly downregulated in the model group, suggesting that *H pylori* breached the mucosal layer and increased the permeability of gastric mucosa. However, Pal promoted the mRNA and protein expression of AJC and Muc1, indicating that Pal could protect the gastric mucosal barrier from the *H.pylori* elicited epithelial structural damage by increasing mucin synthesis and improving epithelial AJC.

Subsequently, UPLC-Q-TOF/MS metabolomic analysis was used to determine the fingerprints of serum and urine metabolic characteristics of GCA rats. PCA and OPLS-DA model showed sufficient sensitivity and specificity to distinguish model from control group. The significant difference between the PalH and model group suggested Pal protected CAG by reversing potential metabolites to normal levels. Meanwhile, the biological function and relevant pathway were identified by HMDB and MetaboAnalyst platform to elucidate the underlying mechanism. 10 related endogenous metabolites were selected as potential biomarkers which were associated with Taurine and hypotaurine metabolism, Glycerophospholipid metabolism, Pentose and glucuronate interconversions, Vitamin B6 metabolism, Pantothenate and CoA biosynthesis, Cysteine and methionine metabolism, Primary bile acid biosynthesis, Arginine and proline metabolism, TCA cycle.

According to the influence value threshold of 0.10, Taurine and hypotaurine metabolism, Glycerophospholipid metabolism, Pentose and glucuronate interconversions are selected as the most relevant pathways. Numerous studies have proved that the disorders of Taurine metabolism play a pivotal pathogenetic role in the initiation and progression of gastrointestinal diseases (Gu et al., 2020). Taurine has been well recognized as an antioxidant both *in vitro* and *in vivo*, and high concentrations of taurine probably contribute to the protection of gastric mucosa from oxidative stress. *H. pylori* infection leads to a decrease in taurine in model rats, indicating that the antioxidant activity decreased in the course of CAG. Glycerophospholipid metabolism was continually disturbed during CAG which has been demonstrated to be closely associated with the immune response. Glycerophospholipid levels are significantly reduced in GCA patients, indicated the disease related dysfunction in glycerophospholipid metabolism (Sun et al., 2020). As one of the subcategories of carbohydrate metabolism, Pentose and glucuronate interconversions is the basis for providing more carbohydrates to the gastrointestinal tract to resist the more complex environment caused by *H. pylori*. In this study, immune dysfunction and energy metabolism disorders together provide a favorable environment for the colonization of *H. pylori*. Pal has a significant callback function on related biomarkers, which may help to improve the CAG induced by *H. pylori*.

Vitamin B6 (VB6) metabolism is necessary to maintain normal physiological functions *in vivo*. Studies have shown that VB6 participates in cellular and humoral immunity during the process of immune response. VB6 deficiency can cause a decline in immunity and affect the expression of certain inflammatory factors (IFN-γ, IL-8), and plays an important role in inflammation caused by gastrointestinal injury (Zhou et al., 2019). As a multifactorial disease, gastrointestinal diseases is ultimately caused by the imbalance between acid secretion and cytoprotective factors. Pantothenate and Pantetheine are important metabolite of Pantothenate and CoA biosynthesis, which are significantly reduced metabolites in gastric ulcers, suggesting Pantothenate and CoA biosynthesis was closely related to the progress of gastrointestinal diseases (Farrokhi et al., 2019). The balance of amino acid metabolism often determines the progression of the disease. In this study, CAG caused an increase in S-adenosylmethionine indicated the imbalance of Cysteine and methionine metabolism and Arginine and proline metabolism. Arginine and proline are considered as potential plasma biomarkers of gastric injury, which can monitor various physiological and pathological conditions of the gastrointestinal tract in real time (Shin et al., 2019). High levels of amino acids is a nutritional requirements for growth of *H. pylori* (Nedenskov, 1994). Turning to another obviously affected amino acid, taurine also affects Primary bile acid biosynthesis. The synthesis and secretion of bile acids are blocked resulting in excessive bacterial growth and impaired epithelial barrier function (AJC and Mcu1), further promoting bacterial transmembrane invasion. It was worth noting that, similar to previous studies, the TCA cycle was down-regulated in this study (Nishiumi et al., 2017). As a critical metabolic checkpoint in the pathological environment, TCA cycle drives a variety of important metabolic pathways and eventually forms a closely related interactive network.

In summary, Pal could reverse the metabolic disorder caused by CAG induced by *H. pylori*, destroy the favorable conditions of *H. pylori* colonization, maintain the gastric mucosal epithelial barrier, restore the immune function of the body, and effectively curb the rapid development of CAG (**Figure 11C**).

## Conclusion

This study was the first to systematically explore the efficacy and molecular mechanism of Pal in the treatment of CAG induced by *H. pylori* based on serum and urine metabolomics. The results showed that the metabolism of taurine and subtaurine, the metabolism of glycerol phospholipid and the mutual transformation of pentose and glucuronide played a dominant role in the Pal treatment of *H. pylori*–induced CAG. The selected metabolites and metabolic pathways might be served as potential drug targets for CAG diagnosis and treatment, which is of great significance for the development of Pal-related drugs for the treatment of CAG.

## Data Availability Statement

The raw data supporting the conclusions of this article will be made available by the authors, without undue reservation, to any qualified researcher.

## Ethics Statement

The animal study was reviewed and approved by Fifth Medical Center of PLA General Hospital (Approval ID: IACUC-2018-010).

## Author Contributions

All authors contributed to the article and approved the submitted version. Conceptualization: XC. Methodology: JZ, RW, HL, CB. Investigation: SW, JW, TY, YW, SR, YT. Writing—original draft: XC. Writing—review and editing: XC. Funding acquisition: YZ.

## Conflict of Interest

The authors declare that the research was conducted in the absence of any commercial or financial relationships that could be construed as a potential conflict of interest.
